# Informational Reinterpretation of the Mechanics Notions and Laws

**DOI:** 10.3390/e22060631

**Published:** 2020-06-07

**Authors:** Edward Bormashenko

**Affiliations:** Chemical Engineering Department, Engineering Faculty, Ariel University, P.O.B. 3, 407000 Ariel, Israel; edward@ariel.ac.il

**Keywords:** Landauer principle, inertial frame of reference, noninertial frame of references, minimal thermal engine, equivalence principle

## Abstract

The informational re-interpretation of the basic laws of the mechanics exploiting the Landauer principle is suggested. When a physical body is in rest or it moves rectilinearly with the constant speed, zero information is transferred; thus, the informational affinity of the rest state and the rectilinear motion with a constant speed is established. Inertial forces may be involved in the erasure/recording of information. The analysis of the minimal Szilard thermal engine as seen from the noninertial frame of references is carried out. The Szilard single-particle minimal thermal engine undergoes isobaric expansion relative to accelerated frame of references, enabling the erasure of 1 bit of information. The energy Δ*Q* spent by the inertial force for the erasure of 1 bit of information is estimated as $\Delta Q≅\frac{5}{3}k_{B}\bar{T}$, which is larger than the Landauer bound but qualitatively is close to it. The informational interpretation of the equivalence principle is proposed: the informational content of the inertial and gravitational masses is the same.

## 1. Introduction

The Landauer Principle, suggesting the thermodynamic equivalent of information, has attracted the interest of researchers in the last decade [1,2,3,4,5]. Simply put, the Landauer principle states that the erasure of one bit of information requires a minimum energy cost equal to $k_{B}Tln2$, where *T* is the temperature of a thermal reservoir used in the process and $k_{B}$ is Boltzmann’s constant [6,7]. Landauer also applied the suggested principle to the transmission of information and reshaped it as follows: an amount of energy equal to $k_{B}Tln2$ (where $k_{B}T$ is the thermal noise per unit bandwidth) is needed to transmit a bit of information, and more if quantized channels are used with photon energies $h\nu >k_{B}T$ [8]. Actually, the Landauer principle converted the information into physical value; Rolph Landauer himself stated that the “information is physical” [7].

The precise meaning, evaluation, and interpretation of the Landauer principle were subjected to intensive scientific discussion [9,10]. The author suggests that the Landauer Principle supports the physical paradigm proposed by J.A. Wheeler, which may be very briefly summarized as follows: “all things physical are information-theoretic in origin”, aphoristically reduced to “it from bit” [11]. Indeed, the Landauer principle enables the estimation of the mass of one bit of information [12] and the establishment of the mass-energy-information equivalence [4,13]. The concept of information has been already successfully exploited for the grounding and clarification of thermodynamics [14,15,16,17,18]. The informational reinterpretation of classical and quantum mechanics is suggested in refs. [19,20,21]. It is assumed in ref. [19] that “the only properties of an electron that we can ever know about are the ones that affect how an electron exerts influence. Another way to think about this is that an electron does not do what it does because it is an electron; rather an electron is an electron because of what it does.” The approach developed in refs. [19,20] avoids the use of empirically defined concepts, such as positions in space and time, mass, energy, or momentum; however, it postulates existence of physical entities that influence one another in a discrete and directed fashion resulting in a partially ordered set of influence events, giving rise to kinematics and dynamics of particles. The “information geometry” introduced in ref. [21] leads to the Hamiltonian appearing in the quantum theory.

The present paper suggests the reinterpretation of mechanics based on the informational approach. An attempt at gaining an informational understanding of gravity is reported in ref. [22], in which gravity is identified with an entropic force caused by the changes in the information associated with the positions of material bodies. The nonrelativistic Schrödinger treatment of the entropy-inspired gravitation is performed in ref. [23]. The approach suggested in ref. [22] and based on the holographic principle is criticized in ref. [24]. The information-based reconsidering of the random molecular motion is undertaken in ref. [25]. The presented paper suggests the alternative informational reinterpretation of basic laws of mechanics.

## 2. Results and Discussion

### 2.1. Informational Reinterpretation of the First Newton Law

Consider the first Newton Law in its traditional wording: “In an inertial frame of reference, an object either remains at rest or continues to move at a constant velocity, unless acted upon by a force” [26]. The First Newton Law was deeply rethought and criticized in the modern era of physics [26]. It was asserted that actually the first Newton Law is not an empirical physical law, but the definition of the inertial frame of reference [26]. Whatever is the precise meaning of the first Newton Law, it establishes the deep physical affinity of the rest and the rectilinear motion with the constant speed. Consider this affinity from the informational point of view. When a particle is in rest or it moves rectilinearly with the constant speed, no information is transferred from one object to another. Indeed, for transferring of at least one bit of the information from a transmitter to a receiver, the particle (it may be photon, electron, or a macroscopic particle) should be emitted and absorbed. Both of these processes necessarily demand the acceleration (deceleration) of a particle [27]. When a particle is in rest or it moves rectilinearly with the constant speed, zero information is transferred. The informational reinterpretation clarifies the affinity of the rest and the rectilinear motion with the constant speed. Moreover, the informational redefinition of the inertial frame of reference becomes possible: “the reference frame of observer for whom an object which does not exchange information with surrounding bodies is in rest or keeps the rectilinear motion with the constant speed is regarded as inertial”.

### 2.2. The Landauer Principle and Noninertial Frames of Reference

**Figure 1:**
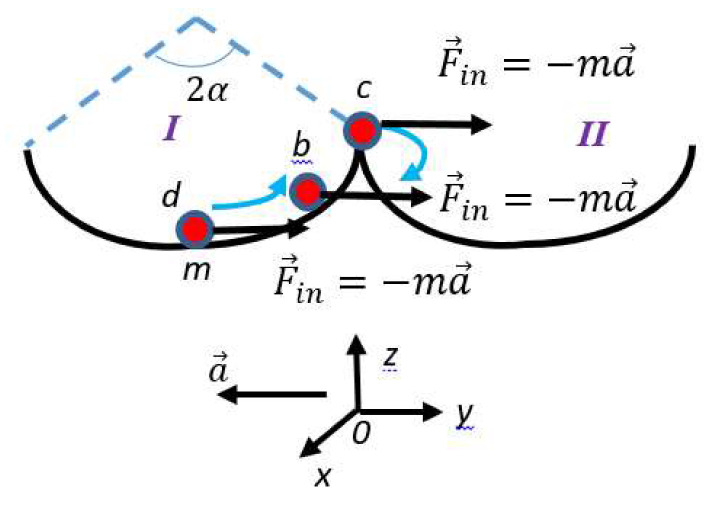
Particle m is placed into the twin-well, symmetrical, frictionless bowl, built of two identical spherical wells labeled “I” and “II”. The initial location of the particle is denoted “a”. Location of the particle in the well labeled “I” corresponds to the informational state “0”; the location of the particle in the well “II” corresponds to the informational state “1”. The inertial force ${\vec{F}}_{in,m}=-m\vec{a}$ transfers the particle from the well “I” to the well “II” (the blue arrow indicates the a→b→c path of the particle, driven by the inertial force).

Now consider the motion of a particle as it is seen from the noninertial frame of reference from the informational point of view. Let us start from the particle *m* confined within a symmetrical double-well potential, which can be stably trapped in either left or right well, corresponding to informational states “0” and “1” [28,29,30]. This may be a particle *m* confined within a symmetrical frictionless bowl, as shown in Figure 1. Location of the particle in the well labeled “*I*” corresponds to the informational state “0”, whereas the location of the particle in the well “*II*” corresponds to the informational state “1”. Initially, particle *m* is in rest in the lowest possible energetic state of the well “*I*”, labeled “*d*” in Figure 1.

Suppose that the bowl is accelerated with an acceleration $\vec{a}$ relative to the particle *m*. In the noninertial frame of references moving with the bowl, the inertial force ${\vec{F}}_{in,m}=-m\vec{a}$ acts on the particle, and if the bowl is frictionless, it will transfer the particle to the well “*II*”, as shown in Figure 1. If the frictionless bowl is spherical, and the angle *α* is given (see Figure 1), the inertia force will transfer the particle *m* to the well “*II*” when an obvious condition $\left|\vec{a}\right|>g\mathrm{tg}\alpha$ holds. Change in the direction of the acceleration will return the particle to the well “*I*”. Thus, it is seen that the inertial force may record/erase information.

For the rough estimation of the thermodynamic cost of erasure on 1 bit of information by the inertial force consider the computing device, exemplified by the single-particle (minimal) thermal engine, suggested by Leo Szilard in 1929 [31,32,33]. This minimal thermal engine is based on a particle *m* enclosed within a chamber (cylinder) divided by half by a partition (piston) *M*, as shown in Figure 1. Finding of the particle *m* in the certain (left or right) half of the chamber corresponds to the recording of 1 bit of information. When the partition is removed, the location of particle is uncertain, and this corresponds to the erasure of 1 bit of information. Location of a particle on the certain half of the chamber corresponds to “1”, and the uncertain location of the particle corresponds to “0”, thus the single-particle-based computer provides the binary logical system [28,29,30,31,32]. The thermodynamic analysis of this computer-engine immediately gives rise to the Landauer bound; namely, the minimal energy necessary for isothermal erasing of one bit of information, which equals $k_{B}Tln2$ [28,29,30,31,32].

**Figure 2:**
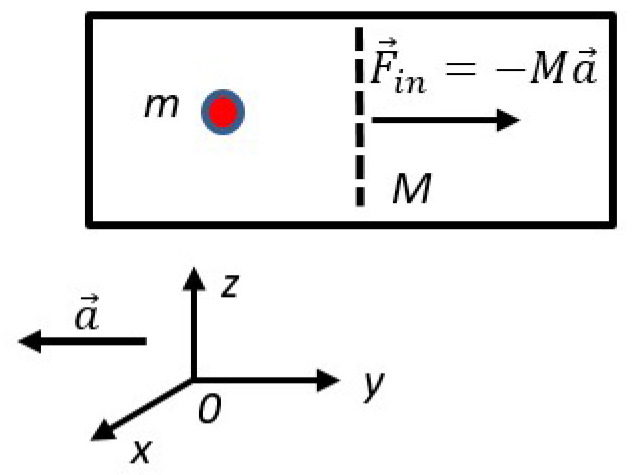
Finding of the particle m in the certain (left or right) half of the chamber corresponds to the recording of 1 bit of information. The partition M is free to slide along the chamber. Frame of references xyz moves with the acceleration $\vec{a}$ relatively to the particle m and the partition M. The inertial forces $-m\vec{a}$ and $-M\vec{a}$ act on the particle m and the partition M. The minimal thermal engine undergoes the isobaric expansion relatively to the frame of references xyz moving with the chamber.

Consider the minimal thermal engine involving the particle *m* and the partition *M*, which may slide in a frictionless way along the chamber, as shown in Figure 2. Address the action of the engine from the noninertial frame of references moving with acceleration $\vec{a}$ relatively to the particle and partition (the chamber remains in rest in the frame of references *XYZ*, as shown in Figure 2). Both the particle *m* and the partition *M* will be exerted to the inertial forces ${\vec{F}}_{in,m}=-m\vec{a}$ and ${\vec{F}}_{in,M}=-M\vec{a}$ [34]. As a result of the motion of the partition exposed to the inertial force ${\vec{F}}_{in,M}$, the location of the particle will become uncertain, and it may be interpreted as an erasure of 1 bit of information. The entire process may be seen as the isobaric expansion of the system under the inertial-force-inspired pressure: $P=\frac{\left|{\vec{F}}_{in,M}\right|}{S}$, where *S* is the cross-section of the partition. The energy ΔQ spent by the inertial force for the isobaric expansion (and erasure of 1 bit of information) is estimated as

(1) $$\Delta Q=\Delta \varepsilon +P\Delta V≅\frac{3}{2}k_{B}\Delta T+P\Delta V≅c_{P}\Delta T≅\frac{5}{2}k_{B}\Delta T=\frac{5}{2}k_{B}T_{1}=\frac{5}{3}k_{B}\bar{T}$$

where Δε is the change in the kinetic energy of the particle, $\Delta V=V_{2}-V_{1}=2V_{1}-V_{1}=V_{1}$ is the change in the volume, occupation by the particle and $\Delta T=T_{2}-T_{1}=2T_{1}-T_{1}=T_{1}$ is the change of its temperature (i.e., an averaged kinetic energy of the particle) due the action of the inertial force ${\vec{F}}_{in,m}$, and $\bar{T}=\frac{T_{1}+T_{2}}{2}=\frac{3}{2}T_{1}$ is the temperature averaged across the isobaric expansion. Equation (1) implies that the motion of the particle is “thermalized” (i.e., randomized), and the notion of the temperature may be introduced for the single-particle system. The conditions of “thermalizing” of small thermodynamic engines are discussed in detail in ref. [33]. Equation (1) actually returns us to within the numerical coefficient to the Landauer bound (consider that the Landauer bound defines the minimal energy necessary for the erasure of 1 bit of information). Of course, the rough estimation supplied by Equation (1) should be taken *cum grano salis*. The use and correct understanding of this estimation is subtle; indeed, the temperature is usually understood as the quantitative measure of the kinetic energy of the random motion of particles [35,36]; whereas, the inertial force gives rise to the directional motion of a particle. Moreover, our treatment was restricted by the case when the chamber was initially divided by the partition by half. More accurate and rigorous treatment was carried out in ref. [28], where the arbitrary initial location of the partition, corresponding to the asymmetric potential wells, used for the thermodynamic analysis of qubit, was considered. This analysis gave rise to the essential correction of the Landauer bound [28]. It was also argued in ref. [28] that an erasure is a process in which the final state of the system is same irrespective of the initial state, for example, if the process starts with the particle in the left/right side of the chamber, finally it will always be in the right side of the chamber; thus, the erasure should necessarily involve the “compression” stage. It is easily seen that the “compression” is attained by reversion of the acceleration $\vec{a}$. Anyway, it is qualitatively demonstrated that inertial forces may be used for the erasure of information within the minimal Szilard-like engine. Reversing the acceleration and correspondingly the inertial force, giving rise to the compression of the particle, may be also exploited for recording of 1 bit of information. It is noteworthy that the inertial forces may be also used for the erasure/recording of information within adiabatically isolated minimal thermal engine [33].

### 2.3. The Informational Reinterpretation of the Equivalence Principle

The understanding of the equivalence principle of general relativity may be ambiguous [27,37]. We adhere to the very tight interpretation of the equivalence principle implying the equivalence of the gravitational and inertial mass $M_{gr}=M_{in}$ [37,38]. Consider inertial mass $M_{in}$ in thermal equilibrium with surrounding (i.e., a thermal bath) under the temperature of *T*. The informational content *I* of the inertial mass $M_{in}$ may be calculated according to the Landauer principles and refs. [4,12,13] according to Equation (2):

(2) $$I=\frac{M_{in}c^{2}}{k_{B}Tln2}=\frac{M_{gr}c^{2}}{k_{B}Tln2}$$

Equation (2) supplies the informational reinterpretation of the equivalence principle, namely, the maximal informational content of the inertial and gravitational masses is the same. It is plausible to assume that the maximal number of bits that may be recorded by a particle in the thermal bath *I* is a relativistic invariant (as well as that the entropy is the relativistic invariant [39]). Thus, Equation (2) implies the relativistic temperature transformation suggested by Ott [40] (for the detailed discussion see refs. [13,39,40]). If the quantum (Bekenstein) restrictions imposed on the maximal amount of information that may be stored in the finite region of space *R* are considered, the informational reinterpretation of the equivalence principle is reshaped as follows [41,42]:

(3) $$I_{max}=\frac{2\pi cRM_{in}}{\hbar ln2}=\frac{2\pi cRM_{gr}\;}{\hbar ln2}$$

Equating expressions (2) and (3) yields

(4a) $$\frac{R}{c}k_{B}T≅\frac{\hbar }{2\pi }$$

(4b) $$\Delta \tau \Delta E\geq \frac{\hbar }{2\pi }$$

where $\Delta \tau ≅\frac{R}{c}$ is uncertainty in the lifetime of the particle confined within the spherical cavity *R*, and $\Delta E≅k_{B}T$ is uncertainty in the thermal energy of the particle. Obviously, Equation (4b) represents the Heizenberg Uncertainty Principle for energy/time with an accuracy to a numerical coefficient.

## 3. Conclusions

John Archibald Wheeler proposed rethinking of basic physical notions and laws within the informational paradigm, aphoristically summarized as “it from bit” [11]. The informational re-interpretation of thermodynamics has been already reported [14,15,16,17,18]. The informational grounding of classical and quantum mechanics was suggested in refs. [19,20,21]. The successful informational reinterpretation of gravity was reported in ref. [22], in which gravity was identified with an entropic force caused by the changes in the information associated with the positions of material objects. The present paper introduces informational reinterpretation of the basic notions and laws of mechanics, namely, the first Newton law and the principle of equivalence. The informational affinity of the rest and the rectilinear motion with a constant speed clarifies the first Newton law. When a physical body is in rest or it moves rectilinearly with the constant speed, zero information is transferred to surrounding physical objects. The informational interpretation of the Einstein general relativity equivalence principle is proposed: the informational content of the inertial and gravitational masses in thermal equilibrium with the surrounding bodies is the same. We demonstrate that the inertial force may be used for erasure/recording of information. The analysis of the action of the minimal Szilard single particle thermal engine as seen from the noninertial frame of references is performed. The Szilard minimal thermal engine undergoes the isobaric expansion relatively to the accelerated frame of references, enabling the erasure of 1 bit of information. The energy ΔQ spent by the inertial force for the erasure of 1 bit of information is estimated roughly as $\Delta Q≅\frac{5}{3}k_{B}\bar{T}$ (where $\bar{T}$ is the temperature averaged across the process) in a qualitative proximity to the Landauer bound.
